# Improving the letters we write: an exploration of doctor–doctor communication in cancer care

**DOI:** 10.1038/sj.bjc.6690374

**Published:** 1999-05-01

**Authors:** D McConnell, P N Butow, M H N Tattersall

**Affiliations:** Medical Psychology Unit, andDepartment of Psychological Medicine, University of Sydney, NSW 2006, Australia; Department of Cancer Medicine, University of Sydney, NSW 2006, Australia

**Keywords:** letters, referrals, cancer consultation, multidisciplinary care, communication

## Abstract

Referral and reply letters are common means by which doctors exchange information pertinent to patient care. Twenty-eight semi-structured interviews were conducted exploring the views of oncologists, referring surgeons and general practitioners. Twenty-seven categories of information in referral letters and 32 in reply letters after a consultation were defined. The letters to and from six medical oncologists relating to 20 consecutive new patients were copied, and their content analysed. Oncologists, surgeons and general practitioners Australia wide were surveyed using questionnaires developed on data obtained above. Only four of 27 categories of referral information appear regularly (in > 50%) in referral letters. Oncologists want most to receive information regarding the patient's medical status, the involvement of other doctors, and any special considerations. Referring surgeons and family doctors identified delay in receiving the consultant's reply letter as of greatest concern, and insufficient detail as relatively common problems. Reply letters include more information regarding patient history/background than the recipients would like. Referring surgeons and family doctors want information regarding the proposed treatment, expected outcomes, and any psychosocial concerns, yet these items are often omitted. Consultants and referring doctors need to review, and modify their letter writing practices. © 1999 Cancer Research Campaign


*British Journal of Cancer* (1999) **80**(3/4), 427–437
© 1999 Cancer Research Campaign
Article no. bjoc.1998.0374

### Improving the letters we write: an exploration of doctor–doctor communication in cancer care

**D McConnell[1], PN Butow[1,2] and MHN Tattersall[3]**

[1]Medical Psychology Unit and [2]Department of Psychological Medicine, University of Sydney, NSW 2006, Australia; [3]Department of Cancer Medicine, University of Sydney, NSW 2006, Australia

**Summary** Referral and reply letters are common means by which doctors exchange information pertinent to patient care. Twenty-eight semi-structured interviews were conducted exploring the views of oncologists, referring surgeons and general practitioners. Twenty-seven categories of information in referral letters and 32 in reply letters after a consultation were defined. The letters to and from six medical oncologists relating to 20 consecutive new patients were copied, and their content analysed. Oncologists, surgeons and general practitioners Australia wide were surveyed using questionnaires developed on data obtained above. Only four of 27 categories of referral information appear regularly (in $>$ 50%) in referral letters. Oncologists want most to receive information regarding the patient's medical status, the involvement of other doctors, and any special considerations. Referring surgeons and family doctors identified delay in receiving the consultant's reply letter as of greatest concern, and insufficient detail as relatively common problems. Reply letters include more information regarding patient history/background than the recipients would like. Referring surgeons and family doctors want information regarding the proposed treatment, expected outcomes, and any psychosocial concerns, yet these items are often omitted. Consultants and referring doctors need to review, and modify their letter writing practices.

**Keywords:** letters; referrals; cancer consultation; multidisciplinary care; communication

Optimal patient care hinges at least in part on adequate and timely exchange of information between treating doctors (Newton et al, 1992). The referral and reply letters are the most common means by which doctors exchange information pertinent to patient care (Tattersall et al, 1995). If these letters meet the respective needs of consultants and referring doctors, discontinuity in care, unnecessary repetition of diagnostic tests and poor patient outcomes such as anxiety, dissatisfaction and loss of confidence in medical practitioners may be avoided (Cummins et al, 1980; McPhee et al, 1984; Hull and Wosterman, 1986; Nutting et al, 1992; Graham, 1994; Epstein, 1995). Few studies have investigated the information content of doctors' letters and/or information preferences of doctor recipients.

Only one study has examined referral letters in the cancer care setting. In this Australian study a limited audit was made of 103 consecutive new patients seen by one radiation oncologist (Graham, 1994). Of the 80 letters available, 95% reported the diagnosis, but only 56% provided a history of the current illness. Less than half the referrals detailed clinical findings or included information on past history, social history, medications and allergies. The author concluded that relevant and important information was not communicated in referral letters.

Only two studies have specifically investigated the content of letters from oncologists, and the information preferences of the recipients. Bado and Williams (1984), in their survey of 73 general practitioners (GPs), reported that technical topics, such as diagnosis, findings on investigation and treatment details, were more important than social topics. More than 80% of GPs, however, wished to receive information regarding the prognosis and what the patient had been told, yet less than 20% of letters adequately covered these topics. The more recent study, conducted in Australia, examined 94 reply letters sent by one oncologist (Tattersall et al, 1995). A questionnaire was sent to 55 GPs and 53 referring specialists who had received a letter from the oncologist asking them to rate each of 14 items as essential, useful, of little use, or of no use.

The majority of respondents ($n$ = 95) rated the following items as essential: diagnosis, clinical findings, test results, further tests, treatment options and recommendations, prognosis, and likely benefits and side-effects. Less than 50% of doctors regarded details of the patients' presenting history, drug or social history as essential. Content analysis of the reply letters found that they usually did not specify prognosis, give recommendations of further tests, or specify the likely side-effects of treatment, and more commonly than referring doctors desired, included details on presenting history, past medical, drug and social history. The extent to which these findings can be generalized, however, is unknown. The letters analysed were from only one oncologist and criteria 'presumed ideal' were used for the content analysis, and to identify doctors' information preferences.

We have conducted a comprehensive audit of referral and reply letters to and from Australian oncologists and explored their information preferences and those of referring doctors (surgeons and GPs). Our objectives were as follows:

- to determine the purpose/function and preferred content of referral and reply letters as perceived by oncologists and referring doctors respectively
- to obtain a representative view of oncologists concerns with referral letters and referring doctors concerns regarding reply letters

*Received 29 June 1998*
*Revised 10 October 1998*
*Accepted 20 November 1998*

*Correspondence to:* MHN Tattersall

**Table 1** What oncologists want in most/all cases and what they get in referral letters

| Content items | Medical oncologists (*n* = 113) | Radiation oncologists (*n* = 43) | Actual content (*n* = 89) % |
|---|---|---|---|
| Factor 1 – Patient's wishes/concerns | Mean = 2.58 | Mean = 3.02[a] | |
| | % most/all | % most/all | |
| How the patient is coping | 48.2 | 63.4 | 6.7 |
| The patient's information, involvement and treatment preferences | 51.8 | 64.3 | 18 |
| Impact of the cancer and treatment on the patient's work, leisure and self-care activities | 37.8 | 56.1 | 6.7 |
| Factor 2 – Patient's background | Mean = 2.61 | Mean = 2.82 | |
| | % | % | |
| Family history of cancer | 39.3 | 52.4 | 3.4 |
| Social history – lifestyle e.g. smoking, drinking | 43.2 | 50 | 10.1 |
| Social history – employment and home situation | 44.2 | 42.9 | 15.7 |
| Clinical/findings on examination | 57.5 | 88.1 | 15.7 |
| Factor 3 – Patient's medical status | Mean = 3.45 | Mean = 3.43 | |
| | % | % | |
| Inter-current medical conditions | **85** | **81** | **22.5** |
| Past medical history | 68.2 | 69 | 20.2 |
| Current medication | **93.8** | **83.3** | **21.3** |
| History of presenting problem | 80.4 | 87.5 | 82 |
| Factor 4 – Involvement of other doctors and their views | Mean = 3.33 | Mean = 3.67[b] | |
| | % | % | |
| Involvement of other doctors in the case | **81.3** | **90.5** | **23.6** |
| Referring doctor's view of his/her continuing involvement in the case | 69.9 | 90.5 | 19.1 |
| What opinions have been expressed by other doctors about patient management | 69.9 | 85.7 | 11.2 |
| What the patient has been told | **80.5** | **90.5** | **13.5** |
| The referring doctor's thoughts on what may be appropriate management | 52.3 | 73.8 | 32.6 |
| Any factors possibly mitigating against particular treatments or treatment arrangements | **85** | **100** | **5.6** |
| Tests/findings on investigation | 98.2 | 100 | 61.8 |
| Factor 5 – Special considerations | Mean = 3.25 | Mean = 3.31 | |
| | % | % | |
| Concerns about psychiatric/social problems | **75.9** | **78** | **3.4** |
| Concerns about patient compliance | 68.8 | 76.2 | 2.2 |
| Concerns about patient understanding | 67.9 | 73.8 | 2.2 |
| Wishes/concerns of the patient's family | 58 | 75.6 | 1.1 |
| Need for an interpreter | **87.4** | **78.6** | **1.1** |
| Information regarding any formal clinical trials the patient is on or is eligible for | 71.4 | 69 | 1.1 |
| Additional items | | | |
| | % Mean | % Mean | |
| Clearly stated reason for referral | 98.2 3.94 | 97.6 3.9 | 78.7% |
| Provisional diagnosis | 88.3 3.51 | 97.6 3.9[b] | 88.8 |
| Copies of test results/reports/films | 94.6 3.85 | 95.2 3.88 | N/A |

Items are listed in order of factor loading. Discrepancies in which > 75% of both medical & radiation oncologists want an item in most/all cases, but < 25% of letters actually contain this item are shown in bold. [a]Denotes a significant difference between mean scores at the level of $P < 0.05$. [b]Denotes a significant difference between mean scores at the level of $P < 0.01$.

- to determine what information is 'typically' contained in referral letters to oncologists, and their reply letters
- to prepare a template of referral and reply letters which may enhance communication between referring doctors and oncologists.

### METHOD

### Stage 1 – qualitative phase

In Stage 1, three medical and three radiation oncologists were invited to participate in an interview and to provide contact details of their last four new patients, their referring doctors and GPs. An invitation to participate was then sent to these doctors. A total of 28 semi-structured interviews with doctors were conducted including seven with oncologists from three Sydney hospitals, ten with surgeons and 11 with GPs practising in the Sydney Metropolitan area. Two interviews were conducted by telephone with GPs in rural areas. All other interviews were conducted in person. The interviews explored doctors' views on referral communications with a focus on their information needs. All interviews were audiotaped and transcribed.

The interview data were analysed using the constant-comparative method proposed by Glaser and Strauss (1967). Put simply, this involves coding each unit of meaning (i.e. specific response), and comparing and contrasting these to identify recurring regularities and discrete categories. This resulted in the development of an analytic framework of 27 discrete categories of information for

 © Cancer Research Campaign 1999

**Table 2** What surgeons and GPs want in most/all cases and what they get in reply letters

| Content items | Surgeons | GPs | Actual content ($n$ = 99) % |
|---|---|---|---|
| Factor 1 – History/background | Mean = 2.53<br>% most/all cases | Mean = 3.03[b]<br>% most/all cases | |
| Lifestyle risk factors | 35.6 | 56.1 | 68.7 |
| Family history of cancer | 40.7 | 70.4 | 66.7 |
| History of presenting problem | **42.6** | 73.6 | **97** |
| Past medical history | **24.6** | 37.9 | **82.8** |
| Social history | 24.1 | 50.4 | 69.7 |
| Current medication | 55.6 | 89.8 | 73.7 |
| Inter-current medical conditions | 59.2 | 69.4 | 64.6 |
| Restatement of reason for referral | 31.5 | **75.7** | 6.1 |
| Factor 2 – Psycho-social concerns | Mean = 3.13<br>% | Mean = 3.61[b]<br>% | |
| Concerns about patient understanding | **64.9** | **84.3** | **6.1** |
| Concerns about psychiatric/social problems | **59.4** | **83.1** | **1** |
| Concerns about patient compliance | 68 | **88.8** | **2** |
| Patient's wishes/expectations regarding information disclosure, decision making/treatment (3) | 66.7 | **86.1** | **26.3** |
| Impact of cancer and/or treatment on patient's work, leisure and self-care activities | 53.7 | **79.6** | **6.1** |
| Likely prognosis (5) | **81.5** | **95.4** | **31.3** |
| How patient is coping/feeling about diagnosis/prognosis/treatment | **68.5** | **87.9** | **16.2** |
| Factor 3 – Examination and investigation findings | Mean = 3.66<br>% | Mean = 3.92[b]<br>% | |
| Tests/findings on investigation | **92.6** | **98.1** | **41.4** |
| Clinical/findings on examination | 74.1 | 95.3 | 89.9 |
| Treatment recommendation | 94.4 | 100 | 85.9 |
| Diagnosis/provisional diagnosis | 86.8 | 100 | 96 |
| Factor 4 – Future management/expectations | Mean = 2.90<br>% | Mean = 3.71[b]<br>% | |
| Likely short- and long-term side-effects | 58.4 | **93.4** | **16.2** |
| Suggestions for management of side-effects | 43.6 | **91.5** | **5.1** |
| Indicators for unscheduled review by the oncologist | 52.8 | **85.8** | **8.1** |
| Aim of treatment e.g. curative or palliative (5) | 81.1 | **97.2** | **40.4** |
| Intention of the oncologist to contact the referring Dr/GP in the future (4) | 54.7 | 87.8 | 51.5 |
| Factor 5 – Treatment/management plan | Mean = 3.57<br>% | Mean = 3.78[a]<br>% | |
| The oncologist's follow-up plan | 90.5 | 98.1 | 67.7 |
| Involvement of other doctors in the case | 80.8 | **89.7** | **32.3** |
| Rationale for recommended treatment (3) | 79.2 | 91.6 | 66.7 |
| Arrangements made for treatment, i.e. where and when | 77.4 | **85.9** | **33.3** |
| What the patient has been told | 88.4 | 92.4 | 49.5 |
| Anything specific the oncologist would like the referring Dr/GP to do. | **92.4** | **99.1** | **14.1** |
| Treatment options | **84.9** | **94.4** | **31.3** |
| Additional item | % Mean | % Mean | |
| Information regarding any formal clinical trial discussed with the patient | **75.5 3.30** | **85 3.55** | **10.1** |

( ) Indicates that the item also loaded on the factor shown in brackets. Items are listed in order of factor loading. Percentage figures shown in bold highlight discrepancies between actual content and preferences of > 50%. [a] = $P < 0.01$; [b] = $P < 0.001$.

referral letters and 32 for consultation reply letters (Tables 1 and 2). Common problems encountered in communication between doctors were identified. This analytic framework was used in Stage 2 to analyse the content of referral and reply letters and provided the basis for the development of questionnaires used in Stage 3 to survey each group of doctors.

### Stage 2 – Content analysis of referral and reply letters

Six medical oncologists from two Sydney hospitals were asked to provide a list of their last 20 consecutive new patients. The patients' medical files were then traced, and referral and reply letters photocopied. During data collection, 21 files were not available and an additional ten referral letters were absent from files. A sample of 89 referral letters and 99 consultation reply letters was therefore obtained.

Most of the referral letters (77%) were from surgeons or other medical specialists, and 93% were outpatient referrals. The content of each letter using the analytic framework developed in Stage 1 was determined by simply noting whether each item of information was present. The first and third author each analysed a random selection of ten letters. Agreement between raters was moderately

© Cancer Research Campaign 1999 

**Table 3** Sample characteristics: Stage 3

| Characteristics | Surgeons | GPs | Oncologists |
|---|---|---|---|
| Sample size | $n$ = 55 | $n$ = 108 | $n$ = 156 |
| Gender | | | |
| Male | 54 (98%) | 65 (60%) | 133 (85%) |
| Female | 1 (2%) | 43 (40%) | 23 (15%) |
| Years of experience | | | |
| Mean | 19.74 | 16.59 | 12.56 |
| Range | 4–40 | 2–50 | 0–39 |
| Speciality | | N/A | N/A |
| General surgeon | 32 (57%) | | |
| Other surgeon | 23 (43%) | | |
| Average number of cancer patients per year | Data not collected | 2 (2%) | Data not collected |
| $<$ 1 | | 33 (31%) | |
| 1–5 | | 27 (25%) | |
| 6–10 | | 45 (42%) | |
| $>$ 10 | | 1 | |

high at 86%, supporting the reliability and utility of the information categories. Upon completion of the coding, nine randomly selected referral and reply letters were recoded to examine intra-rater reliability. A high level of intra-rater agreement was obtained at 98%.

### Stage 3 – survey

In Stage 3, questionnaires for oncologists, surgeons and GPs were developed based on data obtained in Stage 1 (Appendix 1). Oncologists were asked to indicate (a) their preferences for 27 items of information in a referral letter, (b) the frequency with which they encountered seven common difficulties in referral communications and (c) if and when a telephone call was preferred to a letter. Mirroring this, surgeons and GPs were asked (a) their preferences for 32 items of information in letters of reply, (b) the frequency of five common problems in reply letters, and (c) when a telephone call is preferred to a letter. The questionnaires were piloted with three oncologists, surgeons and GPs to ensure clarity in wording and format. The resulting questionnaire was sent to all members of the Medical Oncology Group of the Royal Australasian College of Physicians ($n$ = 148), and all surgeons ($n$ = 84) and radiation oncologists ($n$ = 56) who are members of the Clinical Oncological Society of Australia (COSA). The sample of 200 GPs was drawn from the Directory of Members of the Royal Australian College of General Practitioners which lists almost 10 000 members. The sample of GPs was selected using a randomized block design to ensure a representative proportion from each State and Territory. In total, 113 medical oncologists, 43 radiation oncologists, 55 surgeons and 108 GPs returned completed questionnaires representing a 76%, 77%, 65% and 54% response rate respectively. It was not possible to establish the existence of bias introduced by these response rates which were rather low in the latter two groups. Some demographic details are presented in Table 3.

### RESULTS

### The referral letter – views of oncologists

Analysis of interview data and responses to the survey question concerning the function of the referral letter identified four common themes. The letter (a) provides background information to the patient's situation, and the reason for referral, (b) contributes to assessment by reducing the likelihood of relevant information being overlooked, (c) improves efficiency and quality of care by reducing unnecessary duplication of tests, and providing a focus for history taking, and (d) provides the groundwork for ongoing care and communication. Oncologists reported that missing reports or tests results and insufficient detail in the referral letter were the most frequent concerns and these were more problematic than any other ($P = < 0.05$).

### *Actual vs preferred content of referral letters*

Twenty-seven categories of information sought in referral letters were identified in Stage 1. The questionnaire explored oncologists' preferences for information in new patients referral letters, and respondents indicated on a four-point scale the proportion of cases (none, some, most, or all) in which they would like to receive each of the 27 items of information. The aim was to identify 'in-general' preferences and priorities for information and to examine current practice in light of these.

To identify groups/clusters of items, a factor analysis was undertaken. With oblique rotation, a five-factor resolution emerged, accounting for 51.7% of the variance. Two items, 'reason for referral' and 'provisional diagnosis' did not load on any of the factors above 0.325 and were therefore considered separately in subsequent analyses. Table 1 shows the distribution of the 25 items composing the five factors, the percentage of medical and radiation oncologists wanting each item in most/all cases, and the proportion of letters analysed in Stage 2 in which each item was present.

It is evident that a discrepancy exists between information contained and information desired in referral letters. Only four out of 27 items appear regularly (i.e. in more than 50%) of referral letters, namely, the provisional diagnosis, history of the presenting problem, clearly stated reason for referral and findings on investigation. On these four items only, referral letters appear to meet oncologists' information needs/preferences.

Seven items of information wanted by more than 75% of medical and radiation oncologists in most or all cases were documented in less than 25% of letters. Specifically these items are: (1) inter-current medical conditions, (2) current medication, (3) involvement of other doctors in the patient's care, (4) what the patient has been told, (5) any factors possibly mitigating against particular treatments, (6) concerns about psychiatric/social problems, and (7) need for an interpreter.

In interviews and surveys, oncologists identified circumstances in which their information needs/preferences may very. Several variables relating to individual patient characteristics and the nature of the referral were identified. These variables include: (1) whether the patient is an in-patient or out-patient, (2) whether the doctors interact in a multi-disciplinary clinic, (3) whether the patient is referred preoperatively or post-operatively, (4) whether the cancer problem is simple or complex, (5) how well the referring doctor and oncologist know each other, and (6) whether there are significant psycho-social concerns about the patient. Examining how these variables may affect information needs was beyond the scope of this study, and they are not allowed for in the presentation of preferences which follows.

### *Perceived problems with referral letters*

Oncologists interviewed in Stage 1 identified seven concerns with referral letters (Table 4). In the questionnaire, we asked

© Cancer Research Campaign 1999

**Table 4** Perceived frequency in which each problem with referral letters occurs ($n = 156$)

| Problems – in descending order from most to least frequent | Mean [95% CI] [1 = always, 7 = never] |
|---|---|
| Missing reports/test results – i.e. pathology, X-ray films, operation report | 3.13 (2.94–3.32) |
| Insufficient information and detail in the referral letter | 3.46 (3.26–3.66) |
| No referral letter received prior to or at the time of the consultation | 4.05 (3.82–4.27) |
| Hand-written referral letters which are difficult or impossible to read | 4.19 (3.98–4.41) |
| Unclearly specified reason for referral | 4.89 (4.64–5.14) |
| No referral letter received at all | 5.02 (4.78–5.26) |
| Unnecessary information in the referral letter | 5.70 (5.50–5.89) |

**Table 5** Perceived frequency of problems with reply letters

| | Mean (95% CI) [1 = Always, 7 = Never] | |
|---|---|---|
| **Perceived problems** | **Surgeons** | **GPs** |
| 1. Reply letters arriving late – not promptly | 4.3774 (3.934–4.821) | 3.6944 (3.412–3.977) |
| 2. Unnecessary information in the reply letter | 4.7170 (4.293–5.140) | 5.9907 (5.729–6.252) |
| 3. Insufficient information in the reply letter | 5.3396 (4.985–5.694) | 4.5648 (3.460–5.669) |
| 4. No reply letter received at all | 5.6226 (5.210–6.035) | 4.6262 (4.328–4.925) |
| 5. Letters that are too technical and consequently difficult to comprehend | 6.1887 (5.887–6.490) | 5.8333 (5.577–6.090) |

oncologists to indicate on a seven-point scale the frequency with which each of these seven problems occur (from always to never), and then to identify and rank the three that are most problematic. Mean scores with 95% confidence intervals were computed. Oncologists perceive that missing reports or test results and insufficient detail in the referral letter occur significantly more often than any other problem. These concerns were perceived to be significantly more problematic than any other ($P < 0.05$).

### *Comparison of medical and radiation oncologists*

Figures in Table 1 suggest that radiation oncologists want more information than medical oncologists do in most categories. To statistically explore this finding, the mean score of items in each factor (where 1 = in no cases and 4 = in all cases) were computed separately for each specialty group and compared using *t*-tests for independent samples. Radiation oncologists on average want more information than medical oncologists concerning patients' wishes/concerns ($P < 0.05$) and the involvement of other doctors in the case ($P < 0.01$).

Both medical and radiation oncologists primarily want information regarding the patient's medical status, the involvement of other doctors and special considerations. Information concerning the patient's wishes/concerns and the patient's history/background appear to be of secondary importance.

### *When would oncologists like the referring doctor to phone them?*

Most oncologists (73%) indicated that they would like the referring doctor to phone them (1) when the patient needs an urgent consultation, (2) when there is sensitive information to convey, e.g. if the patient is dissatisfied with other doctors or their management to date, (3) if there are personality or psychological issues that may affect compliance with treatment recommendations, and (4) if the problem is complex and difficult to relate in a letter and multiple opinions have been sought.

### The reply letter – views of referring surgeons and GPs

### *Actual vs preferred content of post-consultation reply letters*

Thirty-two categories of information were identified in Stage 1 as components of post-consultation reply letters from oncologists. In Stage 2, the actual content of the sample of post-consultation reply letters from radiation and medical oncologists were analysed, and in Stage 3, preferences of surgeons and GPs for these items of information were sought. Surgeons and GPs indicated on a four-point scale the proportion of cases (none, some, most, or all) in which they liked to receive letters covering each of the 32 items of information identified in Stage 1. Our aim was to identify 'in-general' preferences and priorities for letter content, and then to evaluate a sample of reply letters with reference to these.

To identify groups of related items, a factor analysis was conducted using the data from the survey of referring surgeons and GPs. With varimax rotation, a five-factor resolution was obtained accounting for 48.4% of the variance. One item failed to load on any factor above 0.325 and was therefore analysed separately, namely, 'information regarding any formal clinical trial discussed with the patient'. The five groups, and items loading are shown in Table 2. Also shown is the percentage of surgeons and GPs

*© Cancer Research Campaign 1999* 

**Table 6** Information prompt sheets

| | |
|---|---|
| | **Referral letters** |
| ® © | Reason for referral |
| ® © | Provisional diagnosis |
| ® © | Succinct history of the problem |
| ® © | Relevant information on patient's medical status – current medications, inter-current medical conditions and relevant past medical history |
| ® | Clinical/findings on examination |
| ® © | Information on tests performed and results |
| ® | Patient's wishes and concerns, e.g. how the patient is coping, and their information, involvement and treatment preferences |
| ® © | What the patient has been told |
| ® © | Involvement of other doctors; what role the referring doctor expects to play; other opinions on management |
| ® © | Any factors possibly mitigating against particular treatments or treatment arrangements |
| ® © | Special considerations, e.g. psychiatric/social problems, concerns regarding compliance or patient understanding, need for an interpreter, and any concerns/wishes of patient's family |
| ® © | Copies of relevant test results/reports |
| | **Reply Letters** |
| ✎ | Restatement of reason for referral |
| ✎ | History of presenting problem, family history of cancer, current medication, intercurrent medical conditions |
| ✄ ✎ | Clinical findings on examination; tests/findings on investigation |
| ✄ ✎ | Diagnosis and likely prognosis |
| ✄ ✎ | Treatment options, treatment recommendation with rationale, treatment aim |
| ✄ ✎ | Patient's wishes and expectations, and how he/she is coping |
| ✄ ✎ | Psycho-social concerns, e.g. patient understanding, psychiatric/social problems |
| ✄ ✎ | Management plan – arrangements, follow-up, and involvement of other doctors |
| ✎ | Likely short- and long-term side-effects, and suggestions for the management of these |
| ✄ ✎ | What the patient has been told |
| ✄ ✎ | How and when to contact the oncologist/consultant |

® = Radiation oncologist, © = Medical oncologist, ✄ = Surgeon, ✎ = GP.

wanting each items in most or all cases, and the percentage of reply letters including each item.

These data suggest that oncologists' letters do not provide all the information surgeons and GPs want. Oncologists' letters commonly provide details on examination and investigation findings (factor 3), and these items are those most often desired by surgeons and GPs. However, the majority of surgeons and GPs want details of the treatment/management plan (factor 5), future management/expectations (factor 4), and psycho-social concerns (factor 2), yet these items are rarely mentioned in letters. Oncologists' letters also frequently detail the patient's background/history (factor 1), which make up six of the ten most common items in reply letters. These items, however, are those least often desired by referring surgeons and GPs.

Several circumstances influencing referring doctor's information preferences were identified. These include: (1) how well the referring doctor knows the oncologist, (2) whether there are routine clinical meetings between the referring doctor and oncologist, (3) the reason for referral – e.g. for second opinion or to take over patient management, (4) whether the patient consultation is pre- or post-surgery, (5) whether the patient is an in-patient or outpatient, (6) whether the cancer is rare or common, and (7) whether the treatment recommended is standard or not. However, examining how these variables may affect information needs/preferences of referring specialists and GPs was beyond the scope of this study and they are not allowed for in the presentation of preferences which follows.

### *Perceived problems with reply letters*

Five potential problems with reply letters were identified in Stage 1 interviews (see Table 4). The surgeons and GPs surveyed indicated on a seven-point scale how often they perceive that each problem occurs, and identified and ranked the three that are most problematic. Mean scores and 95% confidence intervals were computed for each identified problem. Both surgeons and GPs perceive that delay in receiving the reply letter is the most frequently occurring problem, and the problem which is of most concern to them. Superfluous information in the reply letter is perceived by surgeons to be the next most common problem. GPs' however, perceive this to be the least common problem.

### *The preferences of surgeons and GPs for information*

The data in Table 2 suggest that the information needs/preferences of referring surgeons and GPs differ. To test this observation, the mean score of items in each factor (where 1 = in no cases, and 4 = in all cases) for surgeons and GPs were computed and compared using *t*-tests for independent samples. The results indicate that GPs on average want more information than surgeons in every category. Both surgeons and GPs place highest priority on receiving details of the examination and investigation findings, and the proposed treatment/management plan.

### *When would surgeons and GPs like the oncologist to phone them?*

Sixty per cent of surgeons and 78% of GPs identified circumstances in which a reply letter is insufficient and a phone call

 *© Cancer Research Campaign 1999*

desirable. Specific circumstances in which referring doctors would like the oncologist to phone them are (1) when urgent issues arise, (2) when the treatment proposed is unconventional, and (3) when the oncologist is uncertain about the preferred management. A telephone call is also favoured when (4) divergent views exist on treatment approach and (5) if the treatment recommendation is different to that which the referring doctor thought appropriate at the time of referral.

### DISCUSSION

Doctors write many referral letters either to clinical colleagues or to diagnostic service providers. Specialist physicians write letters in reply to referring doctors after new patient consultations or follow-up visits, and to clinicians caring for patients at home following discharge from hospital. Previous studies suggest the content, legibility, speed of receipt and relevance of doctors' letters are often deficient and/or do not meet expectations. We have conducted an information audit of referral and reply letters, interviewed and surveyed a sample of referring doctors and oncologists concerning their preferences and experience with doctors' letters. The results of this study suggest the need for doctors to review, and modify their letter writing practices.

We found that referral letters typically include a statement of the reason for referral, some history of the problem, a provisional diagnosis and description of the findings on investigation. Whilst these items are among the 'most wanted', oncologists in this study have clearly articulated a 'wish' for a range of additional items of information. At the top of oncologists' 'wish list' is information concerning the patient's medical status, the involvement of other doctors and any special considerations. Many oncologists also prefer letters that outline the patient's history and their wishes and concerns, but this information appears to be of secondary importance presumably because these items would be sought during history taking. Radiation oncologists appear to want more information in referral letters than medical oncologists, particularly in the areas of patients' wishes/concerns and the involvement of other doctors. Given the significant discrepancy between information desired and information contained, it is not surprising that oncologists perceive that insufficient information and detail is one of the two most frequently occurring problems with referral letters.

Post-consultation reply letters from oncologists are not meeting the information preferences of referring surgeons and GPs. From the letter writer's perspective, the reply letter also functions as a consultation record. Kamien (1995) has highlighted this dichotomy of purpose, and argued that it must be resolved in the interests of good communication. Should we write two letters, one that is filed in the notes as a record of the consultation, and the second that is prepared specifically to inform the referring doctor and meet their information needs?

Our results confirm previous findings. Tattersall et al (1995) concluded that reply letters, more often than is desired, contain information concerning patient history. This study confirmed that items of information concerning patient history/background are among the most common items in reply letters, but are the least desired. Surgeons and GPs prefer details concerning the treatment/management plan, future management/expectations and psycho-social concerns, yet these are rarely provided in reply letters. There were several items desired by surgeons and GPs in more than 80% of cases, but included in less than 50% of letters. These were findings on examination, details of what the patient has been told, the treatment options, aim of treatment and likely prognosis, the involvement of other doctors in the case, and anything specific the oncologist would like the referring doctor/GP to do. Previous studies have also identified the absence of information on prognosis and what the patient has been told as significant gaps in the information content of 'typical' reply letters.

It is common practice for oncologists to send GPs a copy of the reply letter to the referring surgeon without alteration. Previous studies have either looked at the information needs of GPs alone, or grouped them together with referring specialists. This study compared the information preferences of surgeons and GPs, and our findings suggest that one reply letter may not adequately meet the needs of both. Information preferences appear to be the same, with both surgeons and GPs wanting information concerning examination and investigation findings most, and information regarding patient history/background least. However, the results of this study indicate that GPs want significantly more information than surgeons in every category. These results may explain the differences between surgeons and GPs in their perceptions of problems with reply letters. Superfluous information is perceived by surgeons to be the second most common problem with reply letters. GPs, however, perceive this to be the least frequently occurring problem, if in fact a problem at all.

### Implications for practice

The findings of this study raise doubts as to whether referral and reply letters fulfill their perceived functions. Modifying letter writing practices may be a relatively simple and effective means of improving doctor–doctor communication and hence, patient understanding and outcomes. Referring doctors could improve communication between themselves and medical and radiation oncologists by ensuring that available test results/reports accompany the referral letter, by mailing the referral letter to the oncologist prior to the consultation and giving a copy to the patient. An information prompt sheet for referral letters and letters of reply is provided in Table 6.

Medical and radiation oncologists could take several steps to improve communication with referring surgeons and GPs. Letters should be sent soon after the consultation, since delay in receiving the reply letter is a major concern of both surgeons and GPs. Oncologists' letters should not recount all aspects of the patient history. However, these letters should document the results of examination and investigations, the treatment options and proposed management plan, state the prognosis and what the patient has been told, and outline any psycho-social concerns. Although a case can be made for writing two letters, one for a referring surgeon (if relevant) that is short and succinct, and one for GPs that is more comprehensive, this is clearly not practical. For GPs' standard information sheets may be included with the reply letter concerning the cancer type, potential side-effects of the treatment proposed and recommendations for their management. More than 90% of GPs want this information and less than 20% of oncologist reply letters currently provide any of these details.

### Future research

Future research should examine how the information needs/preferences of oncologists and referring doctors may vary with the circumstances identified in this study. Such research will permit doctors to better predict and tailor their letters to referring and

© Cancer Research Campaign 1999

other doctors. In addition, referral and reply letters, which incorporate the recommendations of this study, should be evaluated to determine whether they result in increased satisfaction on the part of recipients, whether they fulfill their perceived functions as identified in this study and whether they result in better patient outcomes.

### ACKNOWLEDGEMENTS

This study is supported by a NSW Cancer Council Patient Care Award.

### REFERENCES

Bado W and Williams CJ (1984) Usefulness of letters from hospitals to general practitioners. *Br Med J* **288**: 1813–1814
Cummins RO, Smith RW and Inui TS (1980) Communication failure in primary care. Failure of consultants to provide follow-up information. *JAMA* **243**: 1650–1652
Epstein RM (1995) Communication between primary care physicians and consultants. *Arch Family Med* **4**: 403–409
Glaser BG and Strauss AL (1967) *The Discovery of Grounded Theory. Strategies for Qualitative Research*. Aldine: New York
Graham PH (1994) Improving communication with specialists. The case of an oncology clinic. *Med J Aust* **160**: 625–627
Hull FM and Westerman RF (1986) Referral to medical outpatients department at teaching hospitals in Birmingham and Amsterdam. *Br Med J* **293**: 311–314
Kamien M (1995) Writing to referring doctors. *Aust NZ J Med* **25**: 463–464
McPhee SJ, Lo B, Saika GY and Meltzer R (1984) How good is communication between primary care physicians and subspecialty consultants? *Arch Int Med* **144**: 1265–1268
Newton J, Eccles M and Hutchinson A (1992) Communication between general practitioners and consultants: what should their letters contain? *Br Med J* **304**: 821–824
Nutting PA, Franks P and Clancy CM (1992) Referral and consultation in primary care: do we understand what we are doing? *J Family Pract* **35**: 21–23
Tattersall MHN, Griffin A, Dunn SM, Monaghan H, Scatchard K and Butow PN (1995) Writing to referring doctors after a new patient consultation. What is wanted and what was contained in letters from one medical oncologist? *Aust NZ J Med* **25**: 479–482

### APPENDIX 1

The initial patient consultation with a medical or radiation oncologist: doctor and patient information preferences

### QUESTIONNAIRE FOR ONCOLOGISTS

What information do onocologists want to receive with a new patient referral?
What concerns to oncologists have about referral letters?
What are the views of oncologists about providing patients with a post-consultation letter?
This questionnaire is primarily concerned with these three questions. Please take the time (approximately 15 minutes) to fill it in. Your views are important in order to obtain a representative view of oncologists. If you have any questions about this project, please contact Mr David McConnell on (02) 9515 8160.
Your answers will remain strictly confidential. Thank you in advance for your participation.

### Part A – Treatment decision making and working with other doctors

1) In your opinion, how should treatment decisions be made? Please tick the statement which best describes your opinion (please tick one box only)

☐ The doctor should make the decisions based on what he/she determines to be the best treatment for the cancer
☐ The doctor should make the decisions but consider the patient’s priorities and quality of life
☐ The patient and the doctor should make the decisions together
☐ The patient should make the decisions, but consider the doctor’s opinion
☐ The patient should make the decisions using all they know or learn about their treatment options

2) Please indicate the extent to which you agree/disagree with each of the following statements by circling the number which best represents your view (1 = strongly agree, 7 = strongly disagree)

| **Generally speaking**, for patients who may see other doctors… | **Strongly Agree** | | | | | | **Strongly Disagree** |
|---|---|---|---|---|---|---|---|
| A. Oncologists should try to ensure the information they give to patients is compatible with that likely to be given by other doctors | 1 | 2 | 3 | 4 | 5 | 6 | 7 |
| B. Oncologists should consider the views of the patient’s GP in determining the treatment plan | 1 | 2 | 3 | 4 | 5 | 6 | 7 |
| C. Oncologists should consider the views of doctors from other specialities in determining the treatment plan | 1 | 2 | 3 | 4 | 5 | 6 | 7 |
| D. Oncologists should share follow-up with doctors from other specialities | 1 | 2 | 3 | 4 | 5 | 6 | 7 |
| E. Oncologists should share follow-up with the patient’s GP | 1 | 2 | 3 | 4 | 5 | 6 | 7 |
| F. A patient’s cancer care should be jointly managed by the oncologist and the GP | 1 | 2 | 3 | 4 | 5 | 6 | 7 |
| G. A patient’s cancer care should be jointly managed by the oncologist and doctors from other specialities | 1 | 2 | 3 | 4 | 5 | 6 | 7 |
| H. A patient should be referred to an oncologist prior to surgery | 1 | 2 | 3 | 4 | 5 | 6 | 7 |

 © Cancer Research Campaign 1999

**Part B – Information accompanying referrals**

3) In what proportion of cases would you like to receive each item of information listed below in a referral letter?

If you tick *most* or *some* for any item of information, please specify the circumstances in which you want that information from the referring doctor.

| Items of information | With all referrals | With most referrals (specify) | With some referrals (specify) | With no referrals | *Please use this space to specify* |
|---|---|---|---|---|---|
| 1. Patient's social history, e.g. employment, home situation | ☐ | ☐ | ☐ | ☐ | |
| 2. Reason for referral | ☐ | ☐ | ☐ | ☐ | |
| 3. History of presenting problem | ☐ | ☐ | ☐ | ☐ | |
| 4. Family history of cancer | ☐ | ☐ | ☐ | ☐ | |
| 5. Social history – lifestyle, e.g. smoking, drinking | ☐ | ☐ | ☐ | ☐ | |
| 6. Past medical history – unrelated to the presenting problem | ☐ | ☐ | ☐ | ☐ | |
| 7. Inter current medical conditions – physical & psychiatric | ☐ | ☐ | ☐ | ☐ | |
| 8. Current medication | ☐ | ☐ | ☐ | ☐ | |
| 9. Clinical findings: results of physical examination | ☐ | ☐ | ☐ | ☐ | |
| 10. What tests have been done or arranged by the referring doctor & a summary of the main findings | ☐ | ☐ | ☐ | ☐ | |
| 11. Diagnosis/provisional diagnosis | ☐ | ☐ | ☐ | ☐ | |
| 12. Referring doctor's thoughts on what may be appropriate management | ☐ | ☐ | ☐ | ☐ | |
| 13. What other opinions have been expressed by other doctors about patient management | ☐ | ☐ | ☐ | ☐ | |
| 14. Any factors possibly mitigating against certain treatments or treatment arrangements – medical, psycho-social, or demographic | ☐ | ☐ | ☐ | ☐ | |
| 15. Referring doctor's view of his/her continuing involvement in the case | ☐ | ☐ | ☐ | ☐ | |
| 16. Involvement of other doctors in the case | ☐ | ☐ | ☐ | ☐ | |
| 17. What the patient has been told regarding diagnosis, prognosis, treatment options | ☐ | ☐ | ☐ | ☐ | |
| 18. The patient's wishes, expectations or concerns regarding information disclosure, decision making, treatment | ☐ | ☐ | ☐ | ☐ | |
| 19. How the patient is coping and/or feeling about their diagnosis, prognosis or treatment | ☐ | ☐ | ☐ | ☐ | |
| 20. Impact of the cancer & its treatment on the patient's work, leisure and self care activities | ☐ | ☐ | ☐ | ☐ | |
| 21. Any concerns about how much the patient understands | ☐ | ☐ | ☐ | ☐ | |
| 22. Any concerns about psychiatric and/or social problems | ☐ | ☐ | ☐ | ☐ | |
| 23. Any concerns about patient compliance willingness to accept advice | ☐ | ☐ | ☐ | ☐ | |

*© Cancer Research Campaign 1999*

| Items of information | With all referrals | With most referrals (specify) | With some referrals (specify) | With no referrals | Please use this space to specify |
|---|---|---|---|---|---|
| 24. Whether an interpreter is required for the consultation [if the patient has difficulty speaking English] | ☐ | ☐ | ☐ | ☐ | |
| 25. Information regarding any formal clinical trials the patient is on, has been offered, or is eligible for | ☐ | ☐ | ☐ | ☐ | |
| 26. Any wishes/concerns of the patient's family, e.g. about the disclosure of information to the patient | ☐ | ☐ | ☐ | ☐ | |
| 27. Copies of test results, e.g. pathology report, X-ray films | ☐ | ☐ | ☐ | ☐ | |

*Would you like to receive any other information from the referring doctor? If so, please specify on the back of this page.*

4) Is there any information you would prefer to receive over the phone, or circumstances in which you would like the referring doctor to phone you?

☐ Yes ☐ No If yes, please specify.

5) How is the information you receive from the referring doctor helpful? What purpose does it serve?

6) i. You may have experienced the following problems with referral letters. Please circle the number which best represents how often each occurs. (1 = always, 7 = never).

| | **Always** | | | | | | **Never** |
|---|---|---|---|---|---|---|---|
| A. Missing reports/test results – i.e. pathology, X-ray films, operation report etc. | 1 | 2 | 3 | 4 | 5 | 6 | 7 |
| B. Hand-written referral letters which are difficult or impossible to read | 1 | 2 | 3 | 4 | 5 | 6 | 7 |
| C. Unclearly specified reason for referral | 1 | 2 | 3 | 4 | 5 | 6 | 7 |
| D. Insufficient information and detail in the referral letter | 1 | 2 | 3 | 4 | 5 | 6 | 7 |
| E. Unnecessary information in the referral letter | 1 | 2 | 3 | 4 | 5 | 6 | 7 |
| F. No referral letter received prior to or at the time of consultation | 1 | 2 | 3 | 4 | 5 | 6 | 7 |
| G. No referral letter received at all | 1 | 2 | 3 | 4 | 5 | 6 | 7 |
| *Please list any other concerns you may have and indicate how often each occurs* | 1 | 2 | 3 | 4 | 5 | 6 | 7 |
| | 1 | 2 | 3 | 4 | 5 | 6 | 7 |

7) ii. **From the list above**, which 3 concerns about referral letters are most problematic? Please list and rank these with 1 being the most problematic.

1.
2.
3.

8) When would you ideally like to receive the referral letter?

☐ Prior to the patient consultation
☐ At the time of the patient consultation
☐ It doesn't matter

9) In what format would you prefer the referral letter to be written?

☐ In narrative format
☐ In point form
☐ It doesn't matter

© Cancer Research Campaign 1999

10) When a patient is not referred by their GP, how often do you practice each of the following activities? (1 = always, 7 = never)

| | **Always** | | | | | | **Never** |
|---|---|---|---|---|---|---|---|
| i. Send the GP a copy of the letter written to the referring doctor | 1 | 2 | 3 | 4 | 5 | 6 | 7 |
| ii. Write an additional letter to the GP | 1 | 2 | 3 | 4 | 5 | 6 | 7 |
| iii. Send the GP a copy of the letter written to the referring doctor – with an additional post-script | 1 | 2 | 3 | 4 | 5 | 6 | 7 |
| iv. Send the GP a copy of the letter addressed to the referring doctor, but written with the GP in mind | 1 | 2 | 3 | 4 | 5 | 6 | 7 |

### Part C – Patient information

*Several studies suggest that patients have difficulty remembering information conveyed in their initial consultation. We would like to obtain your views on 3 strategies which may address this problem.*

11) A. Do you think patients should be offered **a copy of the letter written to the referring doctor?**

☐ Yes ☐ No ☐ It depends

Please explain:

B. Do you think patients should be offered **an individualized/personal letter** as a follow-up to their consultation with you?

☐ Yes ☐ No ☐ It depends

Please explain:

C. Do you think patients should be offered **an audiotaped recording of their consultation with you**?

☐ Yes ☐ No ☐ It depends

Please explain:

12) Which of the above strategies for providing information do you *most* prefer?

☐ a copy of the letter written to the referring doctor
☐ an individualized/personal letter following their consultation
☐ an audiotaped recording of their consultation
☐ None of the above

13) In your opinion, are there any 'better' strategies (better than those listed above) to ensure that patients are adequately informed?

☐ Yes ☐ No If yes, please specify:

14) In what proportion of cases do you practice each of the following activities? Please tick.

| | **In all cases** | **In most cases** | **In some cases** | **In no cases** |
|---|---|---|---|---|
| i. Dictate your letter to the referring doctor in front of the patient | ☐ | ☐ | ☐ | ☐ |
| ii. Offer patients a copy of the letter written to the referring doctor | ☐ | ☐ | ☐ | ☐ |
| iii. Offer patients an individualized/personal letter after the consultation | ☐ | ☐ | ☐ | ☐ |
| iv. Offer patients an audiotaped recording of the consultation | ☐ | ☐ | ☐ | ☐ |
| v. Offer patients general information booklets | ☐ | ☐ | ☐ | ☐ |

15) Would you like to make any further comments about any of the issues raised in this questionnaire?

*Personal Details*:

16) Your sex ☐ Male ☐ Female

17) Your speciality ☐ Medical Oncology ☐ Radiation Oncology

18) How would you best describe your current position?

☐ University appointment ☐ Visiting Medical Officer
☐ Staff specialist ☐ Private practitioner ☐ Other

19) In what institution is your main practice?

☐ Private hospital ☐ Teaching hospital
☐ District hospital ☐ Other

20) For how many years have you been a practising oncologist? years.

*© Cancer Research Campaign 1999*
